# Microbial Community Structure and Diversity of Endophytic Bacteria and Fungi in the Healthy and Diseased Roots of *Angelica sinensis*, and Identification of Pathogens Causing Root Rot

**DOI:** 10.3390/microorganisms13020417

**Published:** 2025-02-14

**Authors:** Yaya Cheng, Xiaoyun Zhang, Wenwen Zhang, Jianmei Dong, Yanjun Ma, Aimei Zhang, Fujun Han, Hai Peng, Weibao Kong

**Affiliations:** 1 College of Life Sciences, Northwest Normal University, Lanzhou 730070, China; 2022212857@nwnu.edu.cn (Y.C.); 13993119766@163.com (X.Z.); 2023212767@nwnu.edu.cn (W.Z.); 19193112178@163.com (J.D.); mayjdyx@nwnu.edu.com (Y.M.); zhangaimei@nwnu.edu.cn (A.Z.); 2 Institute of Forestry, Fruit and Flower, Gansu Academy of Agricultural Sciences, Lanzhou 730070, China; hanfj2016@gsagr.ac.cn (F.H.); penghai@gsagr.ac.cn (H.P.); 3 Gansu Engineering Research Center of High Value-Added Utilization of Distinctive Agricultural Products, Lanzhou 730070, China

**Keywords:** *Angelica sinensis*, endophytes, microbial community structure, diversity analysis, root rot

## Abstract

*Angelica sinensis* (Oliv.) Diels is an important traditional Chinese herbal medicine, and its main medicinal part is the root. In recent years, root rot has become one of the bottlenecks hindering the healthy and green development of *Angelica* cultivation due to the inappropriate application of chemical fertilizers, pesticides, plant growth regulators, and continuous cropping. In this study, high-throughput sequencing technology was adopted to reveal the differences in the community structure and diversity of endophytic bacteria and fungi in the roots of healthy and diseased *A. sinensis*. The results showed that the diversity index of endophytic bacterial communities was significantly higher in healthy root than in diseased *Angelica* root systems. There was a significant difference in endophytic fungal community diversity only at the m1 sampling site. There was a significant difference in the β-diversity of bacterial communities, but not of fungi. In terms of community composition, Proteobacteria was the dominant phylum of bacteria, and *Sphingobium* and *Pseudomonas* were the dominant genera; Ascomycota and Basidiomycota were the dominant phyla of fungi, and *Plectosphaerella*, *Paraphoma*, and *Fusarium* were the dominant genera. In addition, the relative abundance of the genera *Sphingobium* and *Pseudomonas* was higher in healthy roots, while *Fusarium* was higher in diseased samples. Among the five pathogens isolated from diseased root, four strains were *Fusarium sp.*, and one was *Paraphoma chrysanthemicola*, which is reported for the first time. Our findings indicate that the endophyte community structure of *A. sinensis* infected with root rot changed significantly compared with healthy plants, and *Fusarium* is an important pathogenic factor, which provides a valuable microbiological basis for the targeted biocontrol of *Angelica* root rot.

## 1. Introduction

*Angelica sinensis* is the dried root of *A. sinensis* (Oliv.) Diels, a perennial herb of the Apiaceae family, known for its rich content of volatile oil, polysaccharides, amino acids, and other bioactive compounds [1], and is an important traditional Chinese medicine. *A. sinensis* prefers a cool and humid climate, is suitable for growing in mountainous areas with high altitude, high latitude, and low temperature all year round, and requires loose soil texture and rich organic matter [2]. Owing to the increasing demand for *A. sinensis* in recent years, most *A. sinensis* sold on the market is artificially cultivated. It primarily originates from Min County, renowned as the “Hometown of *A. sinensis* in China” due to its exceptional production and quality, located southeast of Dingxi City, Gansu province. In addition, it is cultivated in Yunnan, Sichuan, Shaanxi, and Hubei provinces [3]. In recent years, the root rot of *A. sinensis* has become a more common problem due to changes in the growing environment and inappropriate planting procedures. The soil-borne infections of *A. sinensis* are caused by fungi, such as *Fusarium*, which is the predominant genus [4]. In addition to reducing *A. sinensis* productivity, root rot deteriorates the plant’s quality, impairs the effectiveness of pharmaceuticals generated from it, and seriously threatens the sustainable development of the *A. sinensis* industry. Symptoms of *Fusarium* root rot include root rot, stem chlorosis, and general leaf wilt [4]. Initially, *A. sinensis* plants suffered from a lack of water; their leaves curled and sagged. In later stages, leaves laid on the ground, and stems and roots turned yellow-brown and gradually decomposed into wet rot [4], emitting the characteristic smell of *Angelica* decay. Eventually, the entire plant withered and died. In contrast, healthy *A. sinensis* plants grow densely, with their leaves pointing upwards. Chemical pesticides have been used for a long time to control *Angelica* root rot. Still, methodological changes have resulted from the potential environmental harm caused by pesticides and their impact on soil microbial imbalance [5]. In recent years, the usage of antagonistic microorganisms and their metabolites has become an alternative to chemical control, offering several advantages such as being environmentally friendly, reducing the risk of chemical residue, and helping to restore soil microbial balance [6].

In recent years, the symbiotic relationship between endophytes and plants has garnered increasing attention from researchers. Endophytes, a type of bacteria that reside within plant tissues without causing noticeable disease, interact closely with their host plants throughout various stages of growth [7]. This symbiosis not only positively influences plant growth and development but also impacts their chemical composition [8,9]. Endophytes are widely found in nature and are typically isolated from surface-disinfected plant tissues without harming the host [10]. Endophytes significantly enhance plant disease resistance through several key mechanisms. First, they produce bioactive substances like antibiotics [11,12] that directly inhibit the growth of pathogens such as *Fusarium* sp. Second, endophytes induce systemic resistance (ISR) [13] in plants, activating defense-related genes and prompting the production of antimicrobial compounds. Third, they compete with pathogens for ecological niches and nutrients, limiting pathogen growth [14]. In summary, endophytes are valuable microbial resources with broad prospects in agricultural production, environmental protection, and biological control.

The primary objectives of this study included the following: a: analysis of differences in endophyte community composition and diversity between healthy and diseased *Angelica* roots using high-throughput sequencing; b: identification of specific *Angelica* root-rot disease-related organisms; c: isolation of microbial taxa and culture of pathogenic bacteria responsible for *Angelica* root rot disease. We combined traditional microbiology and bioinformatics to analyze the experimental data.

## 2. Materials and Methods

### 2.1. Plot Description and Sampling

**Figure 1:**
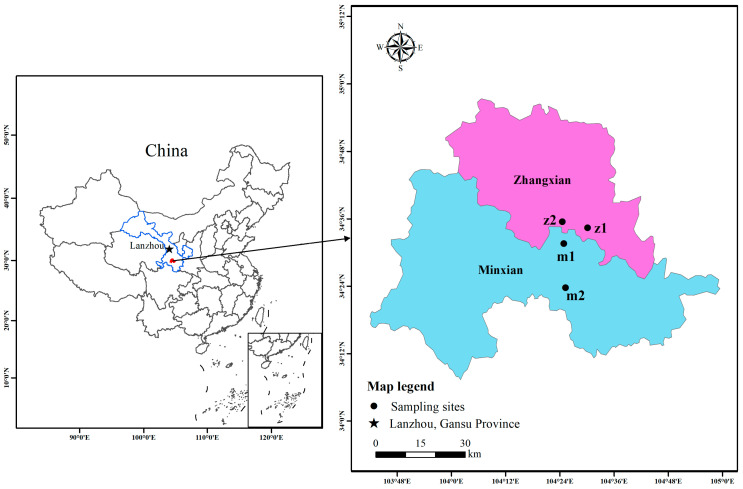
Infographic of sampling sites. Note: m1-h, m2-h, z1-h, and z2-h indicate the samples of healthy plants, and m1-d, m2-d, z1-d, and z2-d indicate the diseased plants.

The sampling sites are situated in Min County and Zhang County of Dingxi City, Gansu Province, located in the southwestern mountainous region of the Loess Plateau in central Gansu Province, at altitudes ranging from 2040 to 3872 m [15]. This area falls within the middle-temperate semi-arid region, characterized by a continental monsoon climate. The annual average temperature is 5.5 °C, and the average annual rainfall is 635 mm [16]. The soil exhibits a loose texture and is notably rich in organic matter. These conditions are highly conducive to the growth of *A. sinensis*. Therefore, four typical *A. sinensis* planting areas were selected as sampling points (Figure 1).

When sampling at each site, the difference between diseased and healthy *A. sinensis* plants should be distinguished. *A. sinensis* plants with the same growth or disease degree were selected. The sampling of diseased and healthy *A. sinensis* plants was conducted with specific spatial considerations to minimize cross-contamination. Diseased plants were selected with a minimum distance of 20 cm between adjacent affected individuals and a gap of more than 50 cm between healthy and diseased plants. Three individuals of both diseased and healthy *A. sinensis* were chosen from each sampling site. Before the excavation of the entire *A. sinensis* plant, the collection rod was meticulously sterilized by applying 75% (*v*/*v*) ethanol, after which the rod was subjected to flame sterilization using a windproof lighter. Each plant was then carefully placed into a labeled ziplock bag and temporarily stored in a foam box containing an ice pack for transportation purposes. Subsequently, the samples were transported to the laboratory and cryopreserved at −80 °C for future experimental analysis.

### 2.2. Sample Pretreatment

The sample pretreatment was based on the method of Herrera [17] with slight modifications. *A. sinensis* samples were initially rinsed with tap water to remove surface sediment. They were then subjected to a sequential washing protocol: rinsing with sterile water for 1 min, immersion in 75% (*v*/*v*) ethanol for 2 min, soaking in a 2.5% (*v*/*v*) NaClO solution containing 0.1% (*v*/*v*) Tween-80 for 5 min, followed by transfer to 75% ethanol for 30 s. Finally, the samples were washed three times with sterile water to ensure the sterility of the plant tissues. After cleaning, the “head” of the root samples was cut into small segments of 0.5–1 cm using sterile scissors and placed in sterile frozen storage tubes labeled and stored at −80 °C.

### 2.3. Illumina NovaSeq 6000 Sequencing

To explore the microbial diversity of *A. sinensis* roots, we performed Illumina (San Diego, CA, USA) MiSeq sequencing. The sequencing of 16S rRNA and ITS gene amplicons in 24 *A. sinensis* root samples was commissioned by Shanghai Personalbio Technology Co., Ltd. (Shanghai, China). Total genomic DNA samples were extracted using the OMEGA Soil DNA Kit (M5635-02) (Omega Bio-Tek, Norcross, GA, USA), following the manufacturer’s instructions, and stored at −20 °C before further analysis. The quantity and quality of extracted DNAs were measured using a NanoDrop NC2000 spectrophotometer (Thermo Fisher Scientific, Waltham, MA, USA) and agarose gel electrophoresis, respectively. The bacterial amplification primers targeted the 16S V5-V7 region (F: 5′-AACMGGATTAGATACCGG-3′, R: 5′-ACGTCATCCCCACCTTCC-3′), while the ITS V1 region was used for fungal amplification (F: 5′-CTTGGTCATTTAGAGGAAGTAA-3′; R: 5′-GCTGCGTTCTTCATCGATGC-3′). PCR amplicons were purified with Vazyme VAHTSTM DNA Clean Beads (Vazyme, Nanjing, China) and quantified using the Quant-iT PicoGreen dsDNA Assay Kit (Invitrogen, Carlsbad, CA, USA). Libraries were constructed using Illumina’s TruSeq Nano DNA LT Library Prep Kit. The Illumina Novaseq 6000 platform was used to conduct paired-end sequencing of community DNA fragments, and the data from the sequencing platform were processed and analyzed.

### 2.4. Bioinformatics Analysis

The diversity of bacterial and fungal microbial communities was analyzed. The DADA2 method [18] of the QIIME2 (v2019.4) software analysis platform was used for sequence denoising or clustering, mainly including priming removal, quality filtering, denoising, stitching, and chimera removal. The length distribution of ASVs was analyzed to identify any abnormal sequences. ASVs were annotated using a pre-trained Naive Bayes classifier with the classify-sklearn algorithm of QIIME2 in the SILVA (bacterial) and UNITE (fungal) databases. Microbial community structures and compositions were visualized. Alpha diversity was assessed using Chao1 [19], Shannon [20], and Simpson index [21]. The Chao1 index was utilized to measure community richness, and the Shannon and Simpson indices were used to evaluate diversity. Beta diversity was analyzed using the UniFrac distance metric, and changes in microbial community structure between samples were visualized via using principal coordinate analysis (PCoA) using R and QIIME2 software.

### 2.5. Pathogen Isolation and Purification

The isolation of pathogens was performed using the tissue isolation method [22]. Initially, diseased *A. sinensis* roots were cleaned with tap water, after which tissue segments were excised and subjected to a surface sterilization protocol, subsequent excision of root tissue at the diseased–healthy junction, approximately 0.5 cm pieces of root tissue. The tissue segments were soaked in 5% NaClO solution for 3 min, rinsed with sterile water, followed by immersion in 75% ethanol for 10 min, and subsequently rinsed again with sterile water. The sterilized tissue segments were then blotted dry using sterile filter papers. Finally, the tissue blocks were placed on PDA (Potato Dextrose Agar) media, with three blocks per dish and three replicates per treatment. After completion, it was placed at 28 °C for cultivation, and when the mycelium grew, the inoculating ring picked up the tip of the mycelium on the PDA for isolation and purification. After isolation and purification, it was stored in a 4 °C refrigerator.

### 2.6. Confirmation of Pathogenicity

*A. sinensis* root tissues were inoculated using the isolated fungi to confirm the pathogenicity of fungi to the plant [23,24]. All five fungal strains isolated from diseased *A. sinensis* roots were tested for pathogenicity, three in each group in parallel. The isolated strain was activated on PDA, punched into a cake of about 6 mm with a hole punch, and set aside. Healthy two-year-old *A. sinensis* plants were initially rinsed with tap water and cut into root segments approximately 6 cm in length. The root segments were then surface-sterilized by soaking in a 5% NaClO solution for 3 min and rinsing with sterile water. Subsequently, the segments were immersed in 75% ethanol for 10 min and rinsed with sterile water. The excess water was removed by blotting it with sterile filter paper. Both ends of the root segments were then wrapped with sterile, skimmed cotton wool and placed on a water agar medium composition. The water agar medium composition was 1 L of distilled water with 15–20 g of agar added for sterilization. The *Angelica* root was cross-scored with a sterile blade. The fungus plug was placed at the cross-wound, and the control group was set as an agar plug without fungus inoculation. Skimmed cotton was moistened with aseptic water to keep it moist, and it was incubated at 28 °C and observed daily to see if any diseased spots were appearing. The re-isolation of the same fungi from the diseased roots proved the pathogenicity of the fungi.

A pot experiment assessed the impact of strain TS, *P. chrysanthemicola*, on the roots of *A. sinensis*. Strain TS was originally isolated from the roots of diseased *A. sinensis*. The *A. sinensis* seedlings were washed with a 75% ethanol solution for 2 min. They were soaked in a 2.5% NaClO solution containing 0.1% Tween-80 for 5 min, followed by a 30 s rinse in 75% ethanol to ensure aseptic conditions. Finally, the plant tissues were washed three times using aseptic water. Subsequently, the sterilized *A. sinensis* seedlings were planted in pots with a diameter of 15 cm and a depth of 20 cm. To maintain the consistency of the soil in the *A. sinensis* planting area, a nutrient-rich substrate with 30% moisture content, incorporating diverse organic matter, was utilized. The plants were watered once every 2 days and inoculated with a TS spore suspension when they reached a height of 8–10 cm. An equal amount of sterile water was used for inoculation in the control group, and after 20 days, the plants were removed from the pots to assess the presence of *A. sinensis* root rot disease. Strain MK, *Fusarium avenaceum*, was used as a typical pathogenic fungus to verify its infectivity on the stem and leaf parts of *A. sinensis* plants, and the methods were consistent with the strain TS pot trial. Strain MK was also initially isolated from the roots of diseased *A. sinensis*.

### 2.7. Molecular Identification of the Pathogens

After 8 days of culture, fungal DNA was extracted using a Rapid Fungi Genomic DNA Isolation Kit (Sangon, Shanghai, China) and amplified by PCR using ITS1 and ITS4 as primers [23]. PCR products were recovered with the AxyPrep DNA Gel Recovery Kit (Axygen, Silicon Valley, CA, USA). The purified PCR products of each strain were taken, and DNA sequencing was carried out using a sequencer, the ABI3730-XL (Applied Biosystems, Foster city, CA, USA). The spliced sequence files were compared with the data in the NCBI nucleic acid database using the NCBI Blast program (https://blast.ncbi.nlm.nih.gov/Blast.cgi, 21 October 2024), and the information of the species with the greatest sequence similarity to the species to be tested was obtained as the identification result. Finally, a phylogenetic evolutionary tree was established using MEGA 11 software.

### 2.8. Statistics and Analysis

For the alpha diversity indices, including Chao1, Shannon, and Simpson, we performed a single-factor analysis of variance (ANOVA) using GraphPad Prism version 10 software. Post hoc tests used Tukey’s Honestly Significant Difference (HSD) method to identify specific group differences when ANOVA indicated a significant effect. The beta diversity was analyzed using the Bray–Curtis distance matrix algorithm. All data are expressed as “mean ± standard deviation”. The visualization process was carried out using QIIME2 (v2019.4), RStudio (R-4.2.2), ggplot2 package, and pheatmap package.

## 3. Results

### 3.1. Microbial Community Analysis in A. sinensis Roots: Composition and Diversity

**Figure 2:**
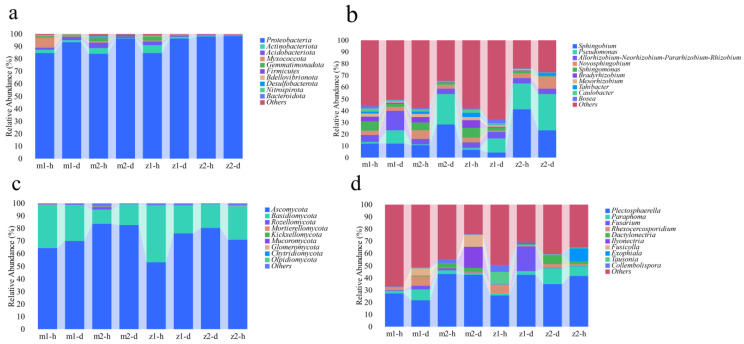
Microbial species composition of *Angelica* root at different sampling sites. Note: (**a**) horizontal relative abundance map of bacterial phylum level; (**b**) relative abundance map of bacteria at genus level; (**c**) horizontal relative abundance map of fungi phylum level; (**d**) relative abundance map of fungi at the genus level. Note: m1-h1 and m1-d1 represent No. 1 healthy *Angelica* sample and No. 1 diseased *Angelica* sample, respectively. The numbering methods of the sampling sites m2, z1, and z2 are the same as those of m1.

This study used high-throughput sequencing technology to analyze the microbial community structure and species diversity of endophytes from the roots of *A. sinensis* at four sampling sites in Dingxi City, Gansu province. The 16S rDNA results showed that a total of 2,134,754 raw data were obtained from 24 samples, and 1,959,774 sequences were obtained after de-noising, with an average of 81,657 sequences per sample and sequence lengths ranging from 372 to 382 bp. The results were clustered into 21,677 bacterial ASVs (Table S1). The dominant bacteria were Proteobacteria, accounting for 84.18–98.37% (Figure 2a), and the dominant genera were *Sphingobium* and *Pseudomonas*. Other genera with an abundance greater than 1% were *Allorhizobium-Neorhizobium-Pararhizobium-Rhizobium*, *Novosphingobium*, *Sphingomonas*, *Bradyrhizobium*, and *Mesorhizobium Tahibacter*, *Caulobacter*, *Bosea*, etc. (Figure 2b). The ITS sequencing results showed that a total of 2,049,068 raw data were obtained from 24 samples. A total of 1,898,157 sequences were obtained after de-noising, averaging 79,090 sequences per sample. The sequence length distribution range was 228 to 361 bp, and the results were clustered into 566 ASVs (Table S2). The dominant fungal group was Ascomycota, accounting for 53.17–83.68%, followed by Basidiomycota, accounting for 11.29–44.75% (Figure 2c). The dominant genera were *Plectosphaerella*, *Paraphoma*, and *Fusarium*. Other genera greater than 1% are *Rhexocercosporidium*, *Dactylonectria*, *Ilyonectria*, *Fusicolla*, *Exophiala*, *Tausonia*, *Collembolispora*, etc. (Figure 2d).

**Figure 3:**
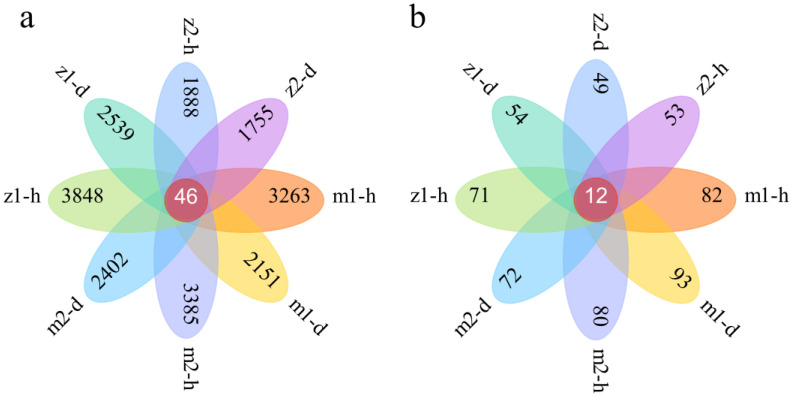
Venn diagram of endophytic bacteria (**a**) and fungi (**b**). Note: m1-h1 and m1-d1 represent No. 1 healthy *Angelica* sample and No. 1 diseased *Angelica* sample, respectively, from Min County sampling site No. 1. The numbering method of m2, z1, and z2 is the same as that of m1.

The Venn diagram provides a visual representation of the microbial composition at the ASV level, highlighting both the shared and unique microorganisms across samples. The ASV number of bacteria in different samples is shown in Figure 3a. A total of 46 ASVs were present in all samples, and the number of ASV in healthy samples at each sampling site was higher than that in diseased samples, indicating high bacterial richness in healthy samples. Figure 3b illustrates the overlap of fungal ASVs across the eight samples collected from the four sampling sites, revealing that there are 12 ASVs common to all samples. Excluding sampling site m1, the healthy samples from the other three sampling sites exhibited more ASVs than the diseased samples, indicating a greater fungal abundance in the healthy samples.

**Figure 4:**
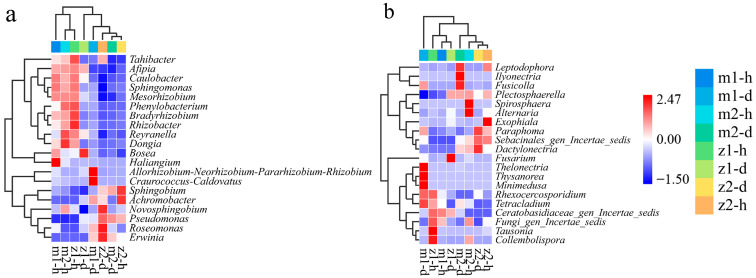
Heat map of genus composition of endophytic bacteria (**a**) and fungi (**b**). Note: m1-h1 and m1-d1 represent No. 1 healthy *Angelica* sample and No. 1 diseased *Angelica* sample, respectively, from Min County sampling site No. 1. The numbering method of m2, z1, and z2 is the same as that of m1.

This experiment used a species composition heat map to compare the differences among samples and display the abundance distribution trends. Figure 4a illustrates the thermal map of bacterial abundance. At the same sampling point, the healthy and diseased groups observed significant differences in endophytic bacteria composition. Taking m1 as an example, high-abundance genera among healthy *Angelica* endophytic bacteria included *Haliangium*, *Caulobacter*, *Sphingomonas*, and *Mesorhizobium*. Conversely, in the diseased group, the bacterial abundance was very low, while certain bacteria absent in the healthy group exhibited high abundance, such as *Allorhizobium-Neorhizobium-Pararhizobium-Rhizobium and Craurococcus-Caldovatus*. Figure 4b illustrates the thermal map depicting the abundance of fungi. At the same sampling point, the abundance of fungi exhibits a similar trend. Using sampling point m1 as an example, *Thelonectria*, *Thysanorea*, *Minimedusa*, and *Rhexocercosporidium* showed high abundance levels. At the same time, the healthy group exhibited very low levels. *Fusarium* was identified as the primary pathogen responsible for *Angelica* root rot. At sampling point z1, *Fusarium* was abundant in the diseased group but almost absent in the healthy group.

### 3.2. Analysis of Alpha Diversity of Endophytes in Angelica Root

**Figure 5:**
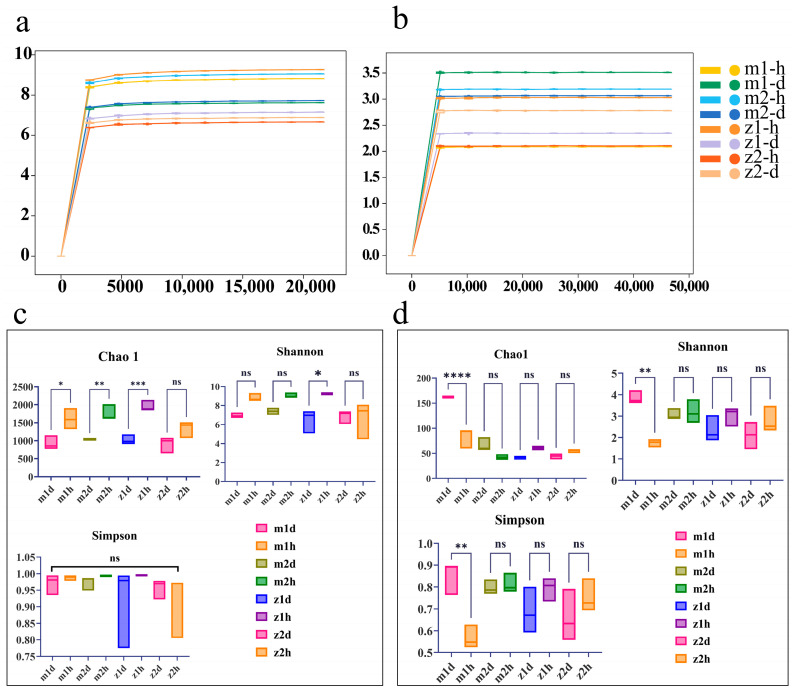
Analysis of alpha diversity and sparse curve of endophytes in *Angelica* root. Note: (**a**) bacterial sparse curve (with sequencing depth on the x-axis and the Shannon diversity index on the y-axis); (**b**) fungal sparsity curve (with sequencing depth on the x-axis and the Shannon diversity index on the y-axis); (**c**) alpha diversity index diagram of bacteria; (**d**) alpha diversity index diagram of fungi. Statistical significance is denoted as follows: *: *p* < 0.05, **: *p* < 0.01, ***: *p* < 0.001, ****: *p* < 0.0001, ns: Not significant (*p* ≥ 0.05).

The Shannon diversity sparsity curve can directly reflect the adequacy of endophytic bacterial sequencing data for healthy and diseased *Angelica* roots. As shown in Figure 5a,b, the curves eventually tend to flatten out, indicating that the sequencing depth was sufficient to capture the microbial diversity in the samples. Figure 5c presents the alpha diversity indices of the bacterial communities across four experimental groups. According to the Chao1, Shannon, and Simpson indices, the bacterial diversity and species richness were greater in roots of healthy *A. sinensis*. Figure 5d illustrates the alpha diversity indices for fungal communities. The findings indicate that, at sampling sites m1 and m2, the Chao1 index for the diseased group surpasses that of the healthy group, implying greater fungal richness in the diseased groups at these sites. Conversely, at sites z1 and z2, the healthy group displays a higher Chao1 index, indicating greater fungal richness in the healthy groups. With the exception of site m1, the Shannon and Simpson indices are consistently higher in the healthy groups, suggesting higher diversity within the endophytic fungal communities of healthy *A. sinensis* roots. However, this trend was reversed at m1, and the Chao1, Simpson, and Shannon indices were significantly higher in the diseased group than in the healthy group at the m1 sampling site.

### 3.3. Relationships Between Microbial Communities in Different Samples

**Figure 6:**
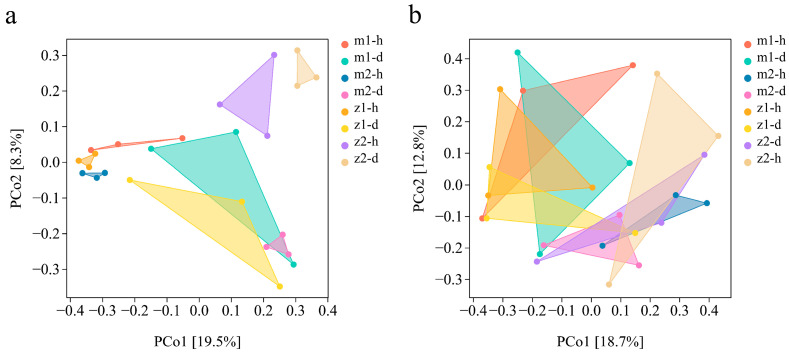
Beta diversity analysis of bacteria (**a**) and fungi (**b**) in the roots of *A. sinensis*. Note: the Bray–Curtis PCoA method. Note: m1-h1 and m1-d1 represent the No. 1 healthy *Angelica* sample and No. 1 diseased *Angelica* sample, respectively, from Min County sampling site No. 1. The numbering methods of sample sites m2, z1, and z2 are the same as those of m1.

Principal coordinate analysis (PCoA) of the communities of prokaryotes (Figure 6a) and eukaryotes (Figure 6b) was performed based on 16S rDNA sequencing and ITS sequencing to reveal the relationships among different samples. The first principal axis (PCo 1) explained 19.5% and 18.7%, respectively. Using PCo 2 as a principal axis, it was possible to accurately show the differences in community structure between different treatment groups by explaining 8.3% and 12.8%, respectively. The findings from 16S rDNA sequencing revealed a total bacterial community variation of 27.8%. *Angelica sinensis* samples from each sampling site exhibited a distinct separation between the healthy and diseased groups, with samples from the z2 site showing greater isolation from the remaining samples. According to the results of ITS sequencing, the fungal community exhibited a total variability of 31.5%. In contrast to the bacterial group, the fungi present at each sampling site were not fully differentiated, a phenomenon hypothesized to be influenced by the proximity of the sampling sites.

### 3.4. Isolation of Pathogens and Confirmation of Pathogenicity

**Figure 7:**
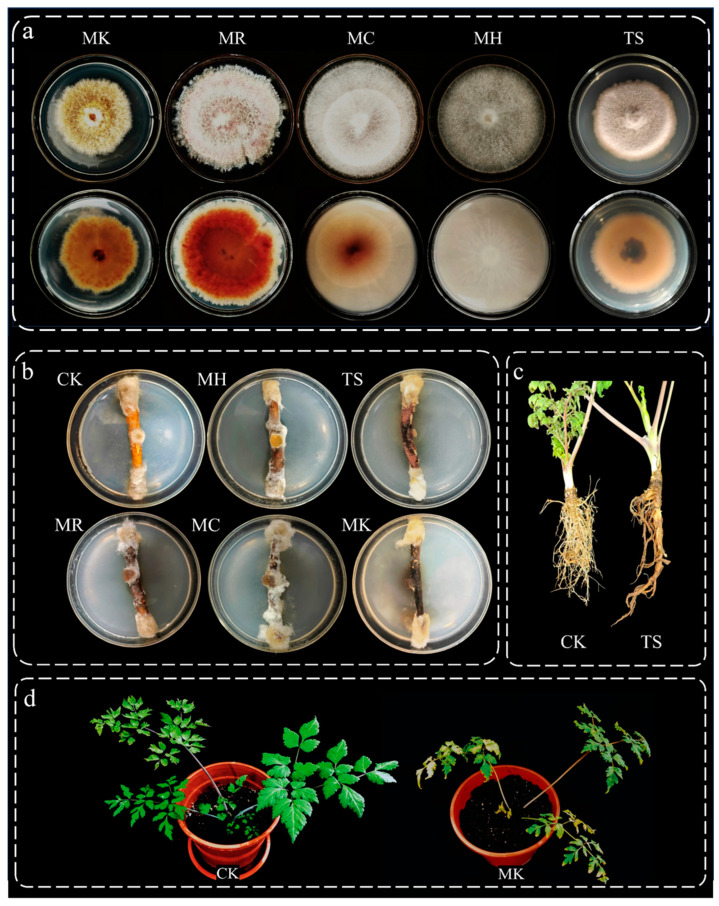
Isolation and pathogenicity testing of pathogenic fungi of *Angelica* root rot. Note: (**a**) five strains of *A. sinensis* root rot pathogens, displaying both the top and bottom views of the culture medium; (**b**) pathogenicity test of pathogens using Koch’s postulates (cultivated for 15 days)—CK refers to the control group using a PDA medium plug that was not inoculated with any microorganisms; (**c**) comparison of roots of healthy and diseased *A. sinensis* in the case of TS—CK indicates the application of sterile water only as a control; (**d**) comparison of healthy and diseased *A. sinensis* plants in the case of MK—CK indicates the application of only sterile water as a control.

After three days of incubation at 28 °C on PDA, filamentous fungi emerged on the cultured tissues. Five distinct fungal strains were isolated and purified using a single-spore isolation technique (Figure 7a) and named MK, MH, MC, MR, and TS according to their origins and characteristics. Pathogenicity tests were conducted using an isolated root inoculation method. The results of these tests indicated that the inoculated sites developed varying degrees of decay, marked by yellow-brown spots, which is consistent with Koch’s postulates. Based on these findings, five strains were identified as the causative pathogens of *Angelica* root rot disease, as shown in Figure 7b. Strain TS was used as a paradigm to observe the effect of pathogenic bacteria on the roots of *Angelica* plants. After inoculation with strain TS, the roots of *A. sinensis* began to rot and soften, producing yellow spots. The fibrous root was significantly reduced compared with the control group (Figure 7c). Strain MK was used as a potential pathogen to observe its pathogenicity on the stem and leaf parts of *A. sinensis* plants. Compared with the control group, the leaves of *A. sinensis* inoculated with strain MK showed brownish-yellow spots, and the plants were short and slow-growing (Figure 7d).

### 3.5. Molecular Biology Identification of the Pathogens

**Figure 8:**
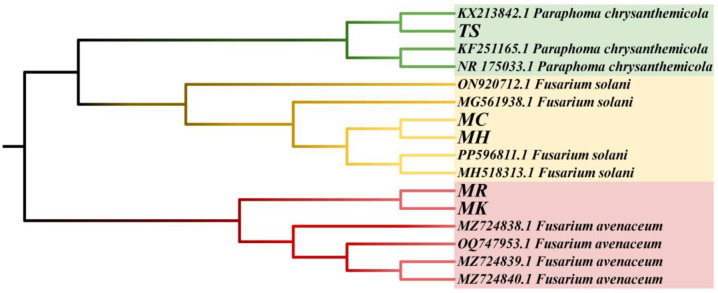
Molecular phylogenetic analysis by the maximum likelihood method.

Using the maximum likelihood method, a phylogenetic tree was constructed for molecular biological identification (Figure 8). Strains MC and MH were identified as *Fusarium solani*, strains MR and MK were identified as *Fusarium avenaceum*, and strain TS was identified as *P. chrysanthemicola*. To our knowledge, this study reported for the first time that *P. chrysanthemicola* is one of the potential pathogenic fungi causing *Angelica* root rot.

## 4. Discussion

Plant growth and development are influenced by microorganisms and disease resistance [25]. At the same time, endophytes are the microbial communities designated to live in the internal tissues of plants and are associated with them [26]. In this study, 21,677 bacteria and 566 fungi were detected through high-throughput sequencing analysis of healthy and diseased *Angelica* root samples. Figure 2 and Figure 3 show that the endophyte communities of healthy and diseased *Angelica* roots were significantly different. Healthy root endophyte communities had a higher diversity index, while diseased ones had a lower diversity index. In addition, some studies have found that disease severity can reduce microbial diversity [27], which is consistent with the results of this experiment. Further observation of Figure 2 shows that when comparing samples from the healthy and diseased groups, the richness of Proteobacteria and *Fusarium* in samples from the diseased group is higher than that of the healthy group. Proteobacteria and *Fusarium* contain many pathogenic microorganisms [28,29]. Therefore, it is hypothesized that certain pathogenic microbes within Proteobacteria and *Fusarium* could be the causative agents of infection in *A. sinensis*.

In this study, we investigated the endophytic bacterial and fungal communities in healthy and diseased roots of *A. sinensis* across different sampling points (m1, m2, z1, and z2). The results showed that the Chao1, Simpson, and Shannon indices were higher in the healthy group, indicating greater bacterial species richness and diversity in healthy roots, which may be due to a more conducive environment for microbial growth. This aligns with previous findings of lower microbial diversity and abundance in diseased plants of *Panax vietnamensis* [30] and higher fungal Shannon diversity index in healthy soils of *Plasmodiophora brassicae* [31]. For fungi, the Chao1 index was higher in the diseased group at m1 and m2 but higher in the healthy group at z1 and z2. The Shannon and Simpson indices were generally higher in the healthy group, except for m1 where only the difference between healthy and diseased groups was significant. This suggests higher endophytic fungal diversity in healthy roots. PCoA analysis revealed that the fungal community structure varied among samples, with 31.5% total variability. Unlike bacterial communities, which showed clear separation in structure among treatment groups, especially at z2, the fungal community was not completely differentiated among sampling sites, possibly due to their proximity. This indicates that the beta diversity of fungal communities differs significantly between sampling points, influenced by location. These findings imply that pathogen infestation affects the structure and diversity of fungal communities in diseased roots.

In terms of species composition, the endophytic community of *Angelica* root was enriched with some beneficial microorganisms, such as *Sphingobium* and *Pseudomonas*. *Sphingobium* and *Pseudomonas* were more abundant in the diseased group than in the healthy group. It is speculated that it may be related to the antagonistic functions of the two bacteria. *Sphingomonas* has various mechanisms of action in assisting phytoremediation, such as promoting bioabsorption, active effluence transport, and reducing toxicity [32]. In addition to nitrogen fixation, phosphorus solubilization, potassium solubilization, and growth promotion, *Sphingomonas* can also act synergistically with other strains to assist in the phytoremediation of heavy metal-contaminated soils [33]. In addition, many *Sphingomonas* strains have been shown to have functions such as nitrogen fixation, phosphate dissolution, and plant growth hormone production, thus promoting plant growth [34] and improving plant stress resistance [35]. *Pseudomonas* is regarded as a model organism for studying beneficial plant–bacteria interaction because *Pseudomonas* can colonize the plant environment well, promote plant growth, and antagonize plant pathogens [36]. *Pseudomonas* can also work synergistically with other biocontrol bacteria, and researchers have used the interaction between *Bacillus subtilis* NCIB3610 and *Pseudomonas viriosa* PCL1606 as a model. The mechanism by which two plant biocontrol bacteria cohabit on the host surface via their distinct regulators was discovered [37]. Our research underscores the complex interplay between the endophytic community and the health of *A. sinensis*. Understanding these interactions is crucial for developing strategies to enhance plant health and disease resistance and optimizing the use of beneficial microorganisms in agricultural practices.

Further analysis revealed the presence of specific flora within the endophytic community of *Angelica* roots that are closely associated with the occurrence of *Angelica* disease. Certain strains of this flora can produce toxic substances that disrupt the *Angelica* growth environment and lead to disease. At the same sampling sites, the abundance of microorganisms in the healthy group was generally higher than that in the diseased group. Conversely, the diseased group exhibited high abundances of *Proteobacteria*, *Fusarium*, and *Plectosphaerella*. The saprophytic ascomycetes in Proteobacteria can cause mold and the decomposition of plant and animal remains in wood, food, cloth, and leather [38]. *Paraphoma* encompasses a variety of pathogens. Some *Fusarium* species inflict damage on cereals and economically important crops, resulting in reduced yields and quality, and produce mycotoxins that pose a threat to human health [39]. Since 2023, ginseng has been reported to suffer from root rot caused by *F. subglutinans*, which leads to the yellowing and wilting of leaves [23]. The presence of pathogens within the endophytic community that are capable of producing harmful substances underscores the necessity for a deeper understanding of these microorganisms and their role in plant health.

Medicinal plants play a crucial role in both traditional and modern healthcare systems due to their therapeutic properties. However, their cultivation is frequently challenged by various biotic stresses, with root rot caused by *Fusarium* being particularly prevalent. This widespread soil fungus inflicts severe root rot on numerous medicinal plants, resulting in substantial yield reductions and quality declines. For instance, Saposhnikovia divaricata experiences significant yield and quality losses due to leaf spot disease caused by *F. acuminatum* [40], while *Gastrodia elata* is afflicted with black rot disease induced by *F. redolens* [16]. Additionally, *F. solani* and *F. avenaceum* strains are known to cause root and stem rot in *Panax ginseng* [41], Ipomoea batatas [42], and *Platycodon grandiflorum* [43]. Notably, *F. avenaceum* has also led to decreased pyrethrum yields in Australia [44], underscoring its detrimental impact on agricultural productivity. In the present study, we identified a strain of *P. chrysanthemicola* that induces brownish-yellow spots on *A. sinensis*, representing the first documented case of root rot caused by this pathogen in China. This finding adds to the existing body of literature, as *P. chrysanthemicola* has previously been reported to cause leaf spot disease in *Atractylodes japonica* [45] and crown and leaf rot in *Atractylodes lancea* [46]. Furthermore, *P. chrysanthemicola* isolated from the roots of *Codonopsis pilosula* was found to possess amylase, cellulase, alkaline protease, and chitosanase activities [47], which may contribute to its pathogenicity in causing root rot in *A. sinensis*. These enzyme activities could potentially disrupt the plant’s cellular structure and nutrient absorption, thereby facilitating the pathogen’s colonization and subsequent disease development. Understanding the mechanisms by which these pathogens affect medicinal plants is essential for developing effective disease management strategies and ensuring the sustainable cultivation of these valuable resources.

## 5. Conclusions

High-throughput sequencing technology was utilized to investigate the endophytic microbial communities in both healthy and diseased roots of *A. sinensis*. This comprehensive analysis identified 566 eukaryotic and 20,077 prokaryotic microorganisms, offering an in-depth profile of the microbial diversity within these samples. The results indicated that the bacterial community in healthy roots exhibited greater diversity and richness compared to the diseased group. Moreover, distinct sampling points revealed varying indices of fungal community diversity. The endophytic community of *A. sinensis* roots was found to be enriched in beneficial microorganisms, such as *Sphingobium* and *Pseudomonas*. In contrast, *Proteobacteria*, *Fusarium*, and *Plectosphaerella* were predominantly identified in samples from diseased roots. Furthermore, five pathogens associated with *Angelica* root rot were isolated from diseased *A. sinensis* roots, including four strains of *Fusarium*, and *P. chrysanthemicola*. In this study, we elucidated the differences in the community characteristics and diversity of endophytes in roots of healthy and diseased *A. sinensis* in the core planting areas of Gansu Province in China. It also provides an important theoretical basis for the exploitation of endophytes resources from *A. sinensis* roots and the prevention and control of root rot.

## Data Availability

The ITS sequencing data have been deposited in the Sequence Read Archive (SRA) under the access number SRP554887. Additionally, the 16S sequencing data are available in the SRA with the access number SRP554892. These datasets are publicly accessible and can be retrieved through the provided access numbers.

## Supplementary Materials

The following supporting information can be downloaded at https://www.mdpi.com/article/10.3390/microorganisms13020417/s1. Table S1: 16S rDNA sequencing results. Table S2: ITS sequencing results.
