# Supplementary material for: Microbial Community Structure and Diversity of Endophytic Bacteria and Fungi in the Healthy and Diseased Roots of Angelica sinensis, and Identification of Pathogens Causing Root Rot
Source: Microorganisms. 2025 Feb 14;13(2):417. doi: 10.3390/microorganisms13020417 (PMC11858288; doi:10.3390/microorganisms13020417)
Supplement: Supplementary file 1 [file microorganisms-13-00417-s001.zip › microorganisms-3332422-supplementary.pdf]

Table S1：16S rDNA sequencing results.

SampleID	Input	Filtered	Denoised	Merged	Non-chimeric	Non-singleton
m1_b1	90287	86850	84653	70372	32490	32430
m1_b2	96438	92537	87682	47532	32468	32367
m1_b3	90036	86364	84778	72678	27726	27697
m1_g1	94637	90787	86960	53188	24734	24602
m1_g2	89451	85690	80387	46331	23784	23579
m1_g3	88959	85081	80700	54873	27031	26893
m2_b1	95482	91749	87498	64651	30655	30506
m2_b2	84666	81141	78525	64202	31570	31499
m2_b3	70390	67705	65420	51410	22593	22514
m2_g1	87654	84105	79277	50331	26872	26685
m2_g2	90190	86192	80752	47686	27170	26977
m2_g3	87273	83597	77106	42248	26799	26571
z1_b1	84596	81010	78802	66313	25609	25557
z1_b2	85933	82413	77707	46836	24063	23905
z1_b3	89245	85750	84354	75898	50295	50265
z1_g1	94250	90401	84967	50730	26445	26237
z1_g2	97247	93719	87416	49474	25259	25049
z1_g3	99365	95966	89353	52471	27389	27167
z2_b1	91770	87777	85081	71470	27871	27811
z2_b2	83637	80275	78903	68783	28927	28896
z2_b3	87615	84109	81768	69158	25688	25642
z2_g1	84992	81708	80543	75124	37514	37502

z2_g2	86693	83223	79543	58710	31729	31613
z2_g3	83948	80341	77599	63074	27044	26966

Note: The sample processing method is DADA2

Table S2: ITS sequencing results.

SampleID	Input	Filtered	Denoised	Merged	Non-chimeric	Non-singleton
m1_b1	64852	60948	60828	59959	55118	55118
m1_b2	151985	136996	136805	135675	135433	135433
m1_b3	70060	65855	65676	65105	56587	56587
m1_g1	63029	58561	58373	57966	55812	55812
m1_g2	61370	52560	52462	50831	48556	48556
m1_g3	66290	60778	60625	58667	56330	56330
m2_b1	66256	62006	61857	60901	57475	57475
m2_b2	69910	64260	64095	62357	57752	57752
m2_b3	75818	71124	70955	70314	67125	67125
m2_g1	69082	65280	65163	64829	61207	61207
m2_g2	63304	59406	59325	59095	56150	56150
m2_g3	72594	67667	67399	66559	59855	59855
z1_b1	121329	115177	115050	114717	105609	105609
z1_b2	84377	77016	76904	74238	71506	71506
z1_b3	94332	86008	85904	85430	80006	80006
z1_g1	86122	78600	78475	76343	72259	72259
z1_g2	80982	72373	72265	71701	69188	69188
z1_g3	102851	94941	94806	93966	84178	84178
z2_b1	94218	89895	89749	89290	82986	82986
z2_b2	91824	87023	86855	86348	80467	80467
z2_b3	105267	99258	99019	98367	91823	91823
z2_g1	85666	81277	81081	80618	74942	74942

z2_g2	113283	105225	104967	104216	97186	97185
z2_g3	94267	89679	89519	89344	86324	86324

Note: The sample processing method is DADA2
